# The Role of Selected Wavelengths of Light in the Activity of Photosystem II in *Gloeobacter violaceus*

**DOI:** 10.3390/ijms22084021

**Published:** 2021-04-13

**Authors:** Monika Kula-Maximenko, Kamil Jan Zieliński, Ireneusz Ślesak

**Affiliations:** The Franciszek Górski Institute of Plant Physiology, Polish Academy of Sciences, Niezapominajek 21, 30-239 Kraków, Poland; m.kula@ifr-pan.edu.pl (M.K.-M.); k.zielinski@ifr-pan.edu.pl (K.J.Z.)

**Keywords:** absorption spectra, cyanobacteria, OJIP-test, phycobilisome, photosynthesis, photosynthetic pigments

## Abstract

*Gloeobacter violaceus* is a cyanobacteria species with a lack of thylakoids, while photosynthetic antennas, i.e., phycobilisomes (PBSs), photosystem II (PSII), and I (PSI), are located in the cytoplasmic membrane. We verified the hypothesis that blue–red (BR) light supplemented with a far-red (FR), ultraviolet A (UVA), and green (G) light can affect the photosynthetic electron transport chain in PSII and explain the differences in the growth of the *G. violaceus* culture. The cyanobacteria were cultured under different light conditions. The largest increase in *G. violaceus* biomass was observed only under BR + FR and BR + G light. Moreover, the shape of the *G. violaceus* cells was modified by the spectrum with the addition of G light. Furthermore, it was found that both the spectral composition of light and age of the cyanobacterial culture affect the different content of phycobiliproteins in the photosynthetic antennas (PBS). Most likely, in cells grown under light conditions with the addition of FR and G light, the average antenna size increased due to the inactivation of some reaction centers in PSII. Moreover, the role of PSI and gloeorhodopsin as supplementary sources of metabolic energy in the *G. violaceus* growth is discussed.

## 1. Introduction

Photosynthetic organisms have specific pigment–protein complexes called antennas that capture light energy and then send excitation energy to the photosynthetic reaction centers, where the primary photochemical reactions occur. Cyanobacteria are believed to be primordial organisms that perform oxygenic photosynthesis and their antenna systems consist of phycobiliproteins, which form the phycobilisome (PBS) complex, located outside of thylakoid membranes [1,2,3,4,5,6]. Cyanobacterial thylakoid membranes contain photosystems I (PSI) and II (PSII), which are universal to all oxyphototrophs but have a lack of typical light-harvesting complexes (LHC) that can only be found in photosynthetically active eukaryotic cells. Instead of LHC, they have PBSs, which contain three main types of phycobiliproteins: (1) phycoerythrin (C-PE), (2) phycocyanin (C-PC), and (3) allophycocyanin (APC). The excited electrons, that are generated by light in the antennas, are transferred to the PS I and PS II reaction centers and initiate the photosynthetic electron transport chain (PETC). Fluorescence emission from chlorophyll *a* (chl *a*) and heat dissipation from PSII, together with PETC, allow for the effective monitoring of changes in the process of photosynthesis in plants, algae, and cyanobacteria [7,8,9]. PBSs absorb light in the wavelength region of 500–650 nm. The displacement of each type of phycobiliproteins in PBS affects the amount of absorbed photon energy. C-PE, which is located at the ends of the antenna, absorbs short wavelengths, i.e.: 498 nm, and in the range from 545 to 575 nm, which have the highest values of photon energy. C-PC absorbs a longer wavelength of light (~620 nm) with a lower photon energy than C-PE, and it is located between C-PE and APC. At the bottom, close to the membrane surface, APC is connected to the protein, which absorbs a wavelength of light ca. 650 nm [3,4,10,11]. 

Cyanobacteria, as with other organisms that perform oxygenic photosynthesis, can maintain efficient conversion of light energy under various environmental conditions. In a similar way to higher plants, cyanobacteria are also able to regulate functions of light-harvesting antennas in response to changes in the environment, such as the spectral composition and intensity of light and/or temperature. Therefore, PBSs perform a major role in these regulatory processes [12,13,14]. The unique feature of aquatic environments is the rapid disappearance of far-red (FR) light with depth compared to terrestrial environments. Despite this, photomorphogenic mechanisms in organisms from aquatic ecosystems are similar to those from terrestrial habitats [15,16]. The vertical distribution of cyanobacteria in aquatic biota depends on the degree of their sensitivity to UV radiation. UV light is a well-known factor that can damage the photosynthetic apparatus of cyanobacteria, and thus, inhibit their growth [17,18,19]. The usage of different white light (WL) wavelength regions by cyanobacteria depends on the ratio of the photosynthetic pigments in the cells, which is a feature of each species and strain. In cyanobacteria, the light is absorbed by chl *a* and PBS, which may suggest a preference for yellow and green light absorbance [20], but not blue and red light, which are absorbed by chl *a* and *b*, as in algae and higher plants [21]. 

The application of traditional light sources, such as fluorescent lamps, incandescent lamps or high-pressure sodium lamps, makes studies that concern the role of specific WL wavelength range in cyanobacteria growth and development very expensive in terms of operation due to their high electricity consumption and heat production. Light-emitting diodes (LEDs) represent an alternative because they are characterized by a much lower energy consumption, long and environmentally friendly functioning [22,23]. LEDs also show excellent regulation of the spectral composition of light due to the use of various chips and wavelength-conversion materials, which allow for adjusting the emitted light to plant and cyanobacteria photoreceptors and enabling the best growth of their biomass [24,25]. In most studies that focus on plant or algae growth, LEDs in the blue light (BL, 400–500 nm), red light (RL, 620–690 nm), and rarely FR light (700–740 nm) region, are used. It is believed that only these colors of the WL spectrum are critical in photosynthesis, phototropism, and photomorphogenic responses [23,26]. The remaining wavelengths of the WL spectrum are omitted because they are considered irrelevant for the proper conditions of the photosynthetic process [23]. 

One of the simplest cyanobacteria is *Gloeobacter violaceus*, frequently used as a model organism in studies related to the origin and evolution of oxygenic photosynthesis [27]. Based on phylogenetic studies with 16S rRNA, it has been shown that *G. violaceus* represents the phylogenetically oldest form among the cyanobacteria [6,28,29,30]. This strain was described by Rippke et al. (1974) on the surface of a limestone rock collected in Canton Obwalden, Switzerland, where higher plants do not grow [31]. Based on the cell morphology and ecology, it was found that *G. violaceus* is similar to *Gloeothece coerulea* cells [32]. The color of *G. violaceus* cells may change to some extent depending on life cycle and environmental conditions [30]. Larger cells, forming aggregates, have a characteristic violet color, which may change to a blue–grey color in suboptimal growth conditions [29,30]. The ultrastructure of *G. violaceus* is distinguished by the absence of thylakoids [6,8,31,33,34] and instead of a typical PBS structure, it contains simpler vertical rods that are associated with the plasma membrane [1,8,10,11,35]. The cell membrane contains photosynthetic apparatus with chlorophyll (chl), various types of carotenoids (car), and phycobiliproteins as antenna pigments [1,36,37,38]. The *G. violaceus* features mentioned above indicate that this strain belongs to the phylogenetically oldest form of cyanobacteria [5,6,30]. 

The growth and PSII activity of *G. violaceus* in different LED-dependent light conditions have not yet been studied. Therefore, the main interest of this work was to verify the hypothesis that some light wavelengths in the range of 390–700 nm affect the PSII antenna and PETC in PSII and lead to differences in the growth of the *G. violaceus* culture. For testing this hypothesis, LEDs were used for the first time as sole effective light sources in the *G. violaceus* culture. It was also found that the amount of phycobiliproteins might perform a key role in the proper functioning of the photosynthetic apparatus of *G. violaceus*. The obtained results allow for approximating the adaptive mechanisms that occur during the growth of *G. violaceus* under different light conditions.

## 2. Results

### 2.1. Growth of the Gloeobacter violaceus Culture Depends on LED Conditions

**Figure 1:**
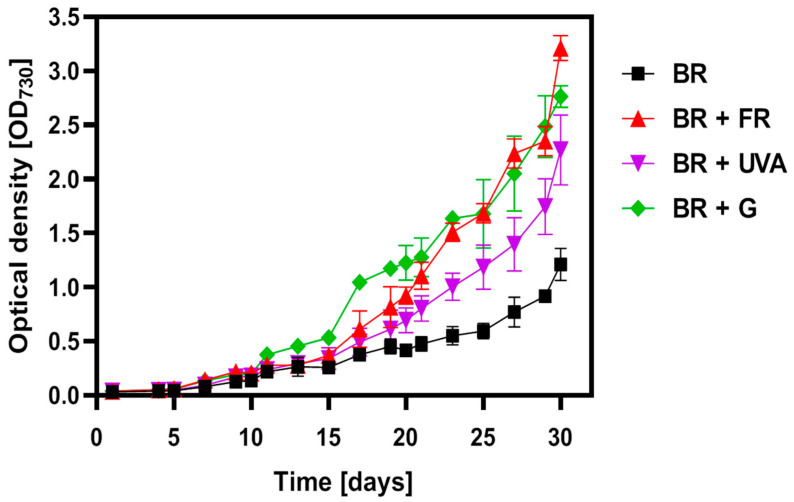
Growth of the *Gloeobacter violaceus* cultures under different light conditions, where the growth of the culture was shown as the increase in the optical density of cells at 730 nm. Light conditions: blue-red (BR) light (black); BR + far-red (FR) (red); BR + ultraviolet A (UVA) (violet); BR + G (green). The values represent means ± SD (*n* = 10).

The weaker intensity of the violet color of the cultures grown under BR light in comparison to other light conditions was related to the proliferation rate of the cyanobacterial cells (Figure 1 and Supplementary Materials Figure S1). 

A lag-induction phase of the *G. violaceus* cultures was observed for all applied lights for the first five days. After the 10th day, the phase of cell growth became exponential and the spectral light composition significantly affected the biomass growth. This effect was especially noticeable in BR light with the additional region of light wavelength, i.e.: BR + FR, BR + UVA, BR + G, where the growth curve showed a typical exponential shape, while for BR light, the shape of the curve was more flattened. The highest increase in biomass was noted in the cultures grown under BR + FR and BR + G light (Figure 1). 

Moreover, it was found that the spectral composition of light significantly influenced the shape of the *G. violaceus* cells grown under the BR + G light (Table 1). The culture grown under the light with the addition of green light (BR + G) was the most elongated, resembling an ellipse-like shape with a diameter of 0.9 µm and a length of 1.5 µm, and was different from the other cultures (Table 1). The cells grown under the other light conditions were more spherical. The BR + FR light induced a larger size but a lower number of cells in comparison to the BR + G light, which stimulated the growth of a larger number of smaller in size and oval cells (Figure 1 and Table 1).

### 2.2. Light Conditions Modify Photosynthetic Pigments Content in Gloeobacter violaceus

**Figure 2:**
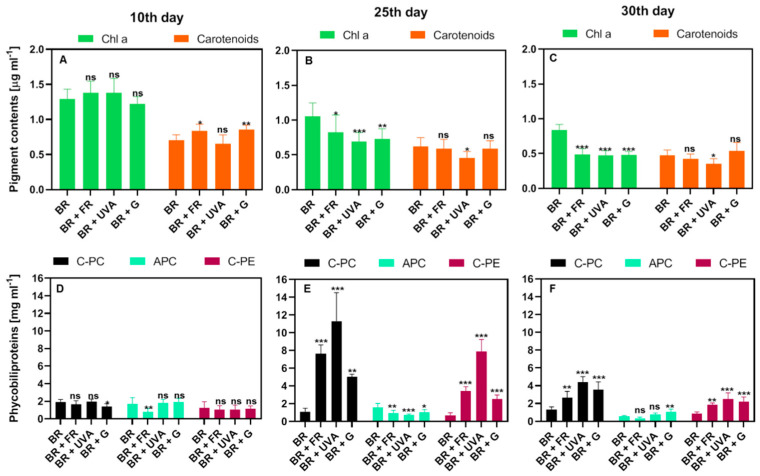
Photosynthetic pigments in *Gloeobacter violaceus* from different days of culture and light conditions. (**A**–**C**) Chlorophyll *a* (chl *a*) and carotenoids content, respectively at 10th, 25th and 30th day of the culture. (**D**–**F**) Content of different phycobiliproteins at 10th, 25th and, 30th day of culture, respectively. C-PC, phycocyanin; APC, allophycocyanin; C-PE, phycoerythrin. The data represent means ± SD (*n* = 12). The significance of the content of the pigments in comparison to the BR (control) was determined based on Tukey’s post-test (*** *p* ≤ 0.0001; ** *p* ≤ 0.001; * *p* ≤ 0.01; ns, non-significant).

In the initial exponential phase of growth (measured on the 10th day), there were no significant differences in the content of chl *a*. However, differences in the levels of car between the cultures grown under various light conditions were observed. Significantly higher amounts of car were found in cells grown under BR + FR and BR + G light compared to in cells grown in BR or BR + UVA (Figure 2A). The content of chl *a* was significantly lower in the cultures grown under BR + FR, BR + UVA, and BR + G light in comparison to BR light. The car content was significantly lower in the cells grown under BR + UVA light as compared to the cells grown under other light conditions (Figure 2B,C). Generally, in the entire growth period, the content of chl *a* and car decreased with the span of the culture, independently of the spectral composition of light.

Differences in the content of phycobiliproteins were found, and they were dependent on the light wavelength. The most visible changes were observed in the content of C-PC and C-PE, which are the main phycobiliproteins of *G. violaceus* [38,39]. The growth phase was not related to the level of APC, while the spectral composition of light affected the APC content (Figure 2D–F). At the beginning of the exponential growth phase, on day 10, the number of cyanobacterial cells was low regardless of the light spectrum. In the cells grown under BR + G and BR + FR light, a significantly lower content of C-PC and APC, respectively, in comparison to other tested light conditions was found (Figure 2D). A distinct increase in the amount of C-PC and C-PE was observed in the cells grown under BR + FR, BR + UVA, and BR + G light on the 25th day of cultivation. However, similar changes were not observed in the cells grown under BR light. Such a large content of the main phycobiliproteins may indicate intensive light harvesting and energy transfer to PSII reaction center (RC), and thus, the achievement of the highest growth rate (Figure 2E). At the end of the exponential growth phase (day 30), there was still significantly more C-PC and C-PE than in the cells grown only under BR light (Figure 2F). The higher content of APC was found in the cells grown under BR + G light in comparison to *G. violaceus* grown under the other spectral regions of used light (Figure 2F). 

**Figure 3:**
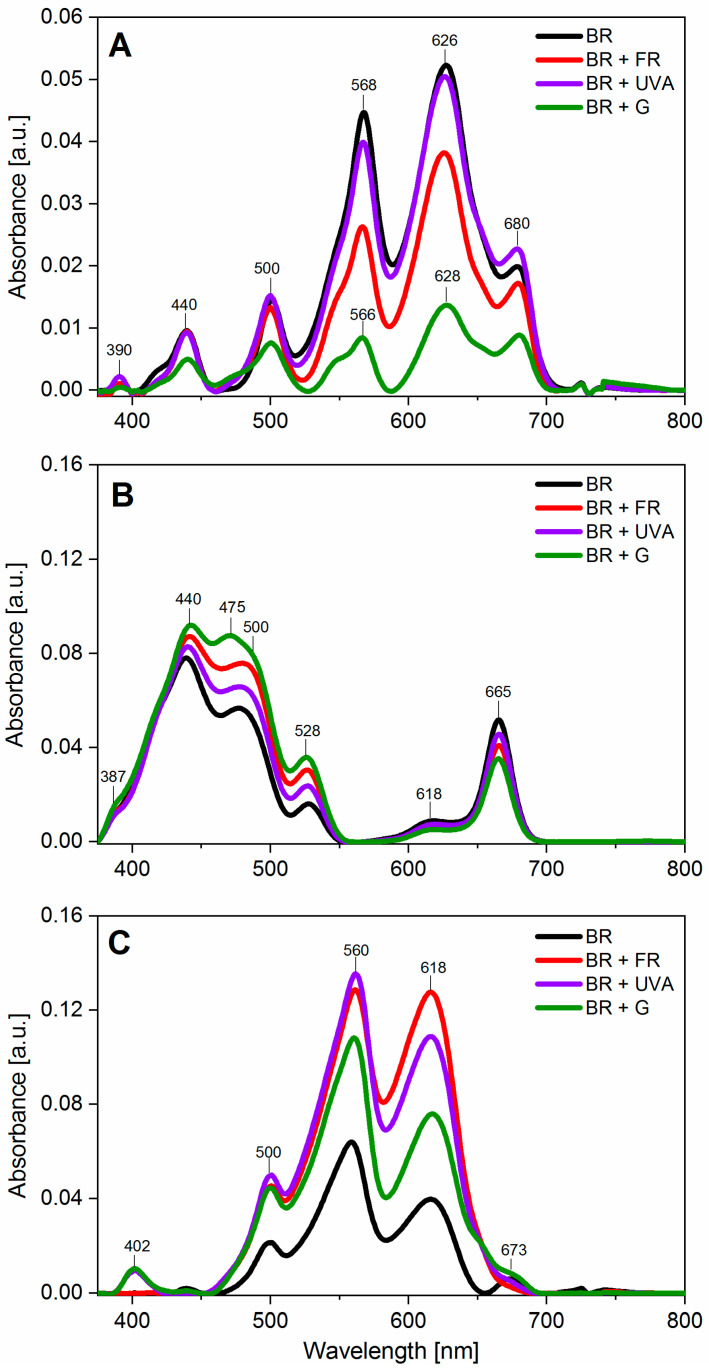
Absorption spectra of the *Gloeobacter violaceus* culture and extracted photosynthetic pigments at the 30th day of culture. (**A**) Absorption spectra of the culture. (**B**) Absorption spectra of chlorophyll *a* and carotenoids. (**C**) Absorption spectra of phycobiliproteins. The main peaks for each spectrum are indicated. The spectra are means from three independent measurements. The lines correspond to the cultures from a definite light spectrum BR (control)—black line; BR + FR—red line; BR + UVA—violet line; BR + G—green line.

The spectrum of live cell cultures in the medium was characterized by higher absorbance intensities ranging from 390 to 680 nm (Figure 3A). The absorption spectra were characterized by six peaks in all cultures. The intensity of these peaks depended on the spectral composition of light used in the culture. These peaks originated from car (390 nm), chl *a* (440 nm), and phycobiliproteins (500, 568 and 618 nm), but the peak at 680 nm was the result of the overlap of the chl *a* and phycobiliprotein bands. The spectra indicated that phycobiliproteins dominated in *G. violaceus*, while chl *a* and car were present to a smaller extent (Figure 3A). *G. violaceus* from BR and BR + UVA light showed a typical absorption spectrum with the predominance of absorbance range for phycobiliproteins, and lower absorbance for the chl *a* and car. The spectrum of the cultures grown under BR + FR and BR + G light was different than for BR. In these spectra, the ratio of the peaks’ intensity for phycobiliproteins of chl *a* and car was much smaller (Figure 3). The content of purified photosynthetic pigments enabled us to identify that the maxima of pigments in cell cultures were dependent on the spectral composition of light (Figure 3B,C). Chl *a* (440 and 665 nm) and car (387, 475, and 528 nm) bands were found in all cultures regardless of the light wavelength used in the *G. violaceus* culture.

In the case of the absorption spectra of the extracted phycobiliproteins, the intensities of the peaks were related to the spectral composition of light. However, independently of the spectral composition of light, in all cultures, there were peaks for phycourobilin (PUB) (500 nm), C-PE (560 nm), C-PC, and APC (618 nm). Moreover, the most likely gloeorhodopsin (GR), with a peak at 402 nm, was identified in the extracts from BR + UVA and BR + G light. A similar spectrum with a peak at 402 nm and 618 nm has been noted for one type of GR with a modified retinal analogue [40]. A peak at 673 nm, corresponding to APC, was visible only in the spectra from BR light, while in other cultures APC was the frame of the peak at 618 nm (Figure 3C).

**Figure 4:**
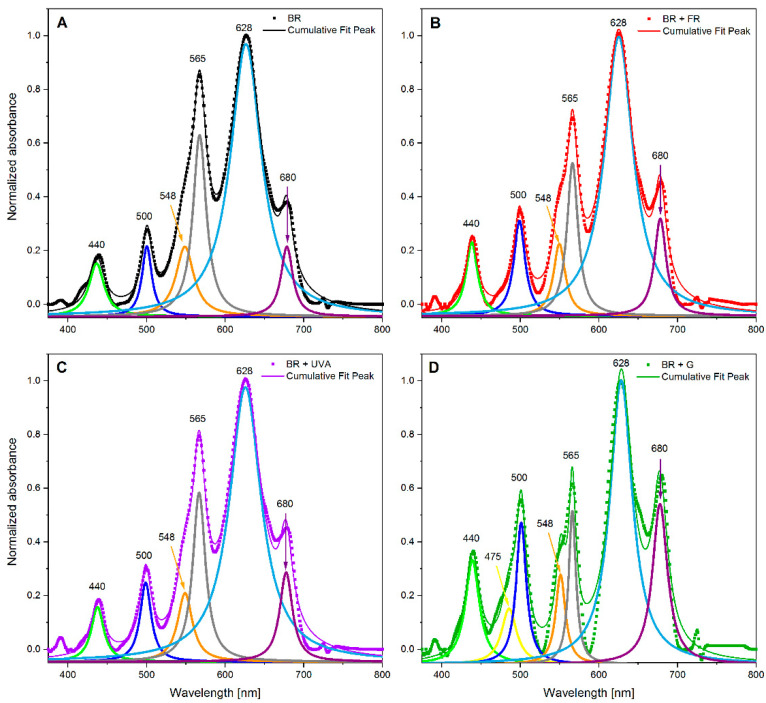
Deconvoluted spectra of the *Gloeobacter violaceus* cultures grown under different light conditions. Solid and dotted lines: BR (control)—black; BR + FR—red; BR + UVA—violet; BR + G—green. Dotted and solid lines describe the original spectra of the culture (as in Figure 3) and cumulative fits after deconvolution (**A**–**D**), respectively.

For a more detailed analysis that concerns the occurrence of individual photosynthetic pigments, a deconvolution of the spectrum of the *G. violaceus* cultures from experimental light conditions was performed. As a result, the absorption spectrum of each of the cyanobacterial cultures was divided into six peaks characteristic to individual photosynthetic pigments (Figure 4). Moreover, an additional peak at 475 nm was identified in the spectrum of the culture grown under BR + G light (Figure 4D). The locations of six peaks were common to all tested cultures, regardless of the spectral composition of the light. However, their intensity was related to the light conditions of the culture growth (Figure 4). The identified peaks and their area are presented in Table 2. It was found that C-PC (peak at 628 nm) was the most abundant pigment in *G. violaceus* cells. However, the C-PC content in the cells grown under the BR + G light was much lower than in cyanobacteria from other cultures. It was noted that the increased contents of C-PE and GR pigments were in the culture grown under BR light, compared to cyanobacteria from BR + FR, BR + UVA, and BR + G light conditions. On the other hand, adding another wavelength to BR light increased the amount of R-phycoerythrin (R-PE), car, and GR if compared to their content under BR light only. 

### 2.3. PSII Activity in Gloeobacter violaceus Is Affected by the Spectral Region of Light

#### 2.3.1. OJIP Test

The fluorescence curve course (Vt) may allow for the evaluation of both photochemical and thermal phases of photosynthesis [43,44,45]. We can distinguish five main phases: the O-K, O-J, J-I, and I-P phase. In general, the curve shape course for *G. violaceus* was very similar to that observed for other cyanobacteria, algae, and higher plant leaves [46,47,48,49]. 

**Figure 5:**
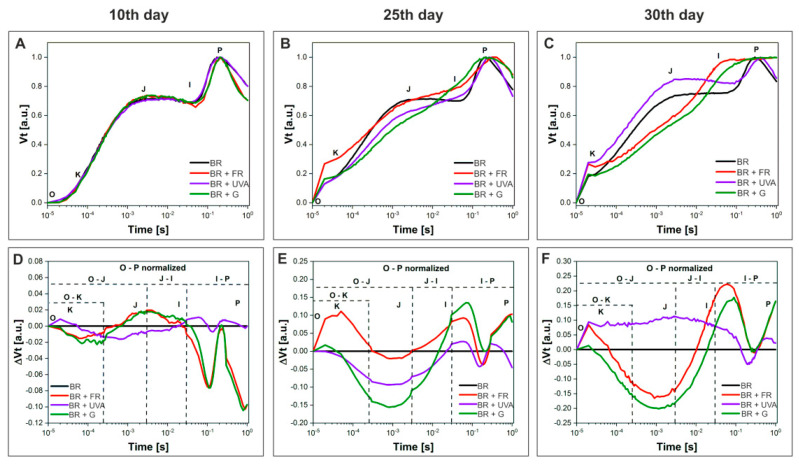
The effect of spectral light compositions on *Gloeobacter violaceus* photosystem II (PSII) activity at 10th (**A**,**D**), 25th (**B**,**E**) and 30th (**C**,**F**) day of culture, respectively, grown under different light conditions: BR (control)—black line; BR + FR—red line; BR + UVA—violet line; BR + G—green line. (**A**–**C**) Chlorophyll *a* fluorescence induction curves (ΔVt). The K point indicates the K step at about 300 μs, the J point indicates the J step at 3 ms, the I point indicates the I step at 30 ms, and the P point indicates the P step at about 300 ms. (**D**–**F**) ΔVt curves represent the difference of variable fluorescence curves, which were calculated as the difference between the normalized fluorescence curves for a control curve (BR), that was equal to “0” (ΔVt = VtBR − VtBR = 0) and BR + FR, BR + UVB, and BR + G. The presented data are mean values from 8 independent replicates.

**Figure 6:**
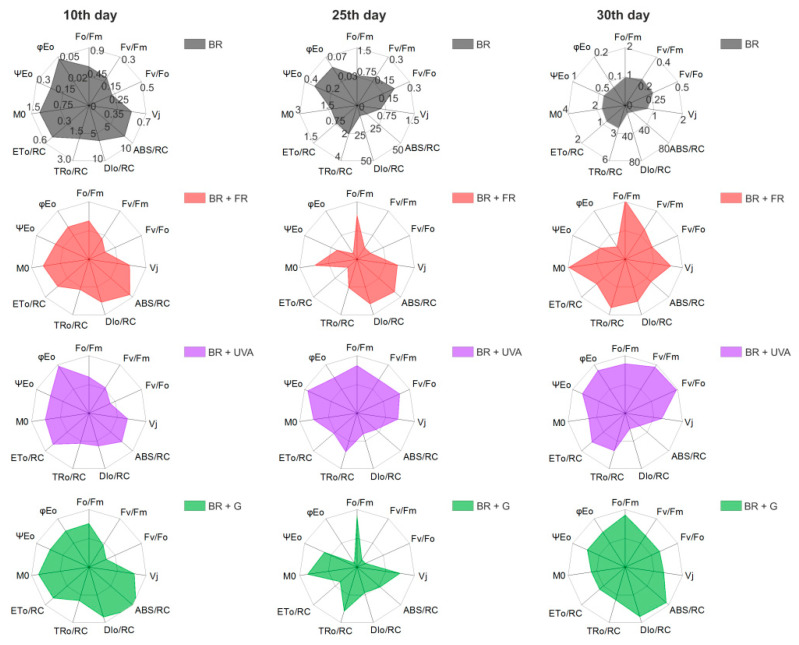
The spider-plot presentation of selected fluorescence parameters quantifying the changes of PSII of *Gloeobacter violaceus* from different days of culture and light conditions. The presented data are mean values from 8 independent replicates. The fluorescence parameters are described in Table 3 (Materials and Methods).

Based on the changes in the OJIP-test curves and the parameters of the kinetics of chl *a* fluorescence of cyanobacterial cultures, the comparison of changes in the flow of electrons between PSII and PSI in different phases of growth, as well as in terms of the spectral composition of light was performed. This allowed for evaluating the activity of the photosynthetic apparatus in *G. violaceus*. The differences in the electron transport efficiency (I-P curve, ψEo) and the number of electrons on the acceptor side of PSII (J-I curve, φEo), dependent on the growth phase and spectral composition of light, were observed (Figure 5 and Figure 6).

The decrease in the number of electrons transported from quinone A (Q_A_) to quinone B (Q_B_) on the acceptor side of PSII, observed as an increase in the J-I curve, was found in the cultures grown under BR + FR and BR + G light during the adaptation phase (day 10). This indicates a decrease in the electron flow from PSII to PSI. Moreover, the relationship is shown as positive values on the ΔVt plots in the J-I phase (Figure 5D). In the exponential phase of growth (day 25), there was an increase in the electron transport efficiency in the cultures grown under BR + FR, BR + UVA, and BR + G light compared to the cultures grown under BR light, while the electron transport was still reduced in cyanobacteria grown under BR + FR light (Figure 5E). In the last stationary phase of growth (day 30), in the culture grown under BR + UVA light, a reduction in electron flow was observed, i.e., thus, the efficiency of electron transport from PSII to PSI decreased compared to the exponential phase of growth of the cultures from other light conditions (Figure 5F). The cyanobacteria from BR + FR and BR + G light showed the highest efficiency of PETC. 

The I-P phase on the ΔVt curve showed large differences in the course of the curve depending on the phase of growth and the spectral composition of light (Figure 5D–F). In phase I-P, the negative values of ΔVt indicate more NADP + molecules per active RC, and an increased pool of plastoquinone (PQ). This result suggests a compensatory mechanism in response to altered light conditions. Positive values of ΔVt in the I-P phase were found in the cultures grown under BR + FR, BR + UVA, and BR + G light, and were slightly decreased under BR + UVA light (Figure 5E,F). This implicates a reduced pool of PQ and suggests an adaptation of *G. violaceus* to light conditions, which in turn contributed to more efficient photosynthesis that led to a greater increase in biomass in the BR + FR, BR + G, and BR + UVA cultures (Figure 1). Moreover, it should be noted that the appearance of the K point on the ΔVt curve in the O-K phase indicates a disturbance in the oxygen-evolving complex and an occurrence of stress conditions [46,50]. It is worth emphasizing that the K point appeared in the culture grown under BR + FR light only for the exponential growth phase (Figure 5E,F), when the culture showed a large increase in biomass (Figure 1).

The determined fluorescence parameters describing the quantum yield and energy flow through the RC showed significant differences in the functioning of the photosynthetic apparatus of *G. violaceus* depending on the phase of growth and the light growth conditions. Most determined fluorescent parameters for cyanobacteria cultured in BR light (control), in days 10, 25, and 30, were increased, which indicates the induction of PSII efficiency in *G. violaceus* (Figure 6 and Supplementary Materials Table S1). Elevated activity of PSII in BR light was also reflected in the biomass increase. 

It should be noted that the majority of values of the measured parameters increased with the proliferation of cyanobacteria in the cultures (Figure 1). On the 10th day of culture, a decrease in the values of Fv/Fm, Fv/Fo, φEo parameters in the cells grown under the BR + FR and BR + G light was found compared to the values for the cells grown under the BR light only. Aside from that, on day 25 and 30, the values of Fo/Fm and M0 increased in the cells grown under BR + FR and BR + G light (for BR + G only in day 25) in comparison to BR light (control). The increase in these parameters indicates a disturbance of the PSII efficiency and the occurrence of stress in these cultures already in the adaptation phase of growth (day 10) (Figure 6). In the exponential phase of growth (day 25), multiple changes in PSII functioning were found in the cultures grown under BR + FR, BR + UVA, and BR + G light, and they affected the increase in the biomass of these cultures. In the exponential phase of growth, the values of the Fo/Fm, Vj, M0 parameters increased, while the value of quantum electron transport efficiency (φEo) decreased in the cultures from BR + FR and BR + G light in comparison to the control (Figure 6). Moreover, there was a decrease in the values of the Fv/Fm and Fv/Fo parameters in cells grown under BR + FR and BR + G light. A higher index of the rate of closure of RCs (Vj), an increase in the net rate of Q_A_ reduction (M0) as well as the major dissipation of non-captured energy as heat and/or fluorescence (DIo/RC), indicate altered PSII performance in these cultures. Despite the disturbance of PSII functioning, these cultures were characterized by a large increase in biomass, which may indicate the operation of a different compensation process or the choice of a different way of electron transport than by PSII (day 25) (Figure 6). In the stationary phase of growth (day 30) under BR + FR, BR + UVA, and BR + G light, a significant increase in the biomass of *G. violaceus* cultures was observed, which was also related to the elevated values of most of the analyzed fluorescent parameters in comparison to the culture grown under BR light. Only the value of φEo was unchanged in the culture grown in BR + FR light compared to the other cultures (day 30) (Figure 6).

#### 2.3.2. The Energy Flux Pipeline Model

**Figure 7:**
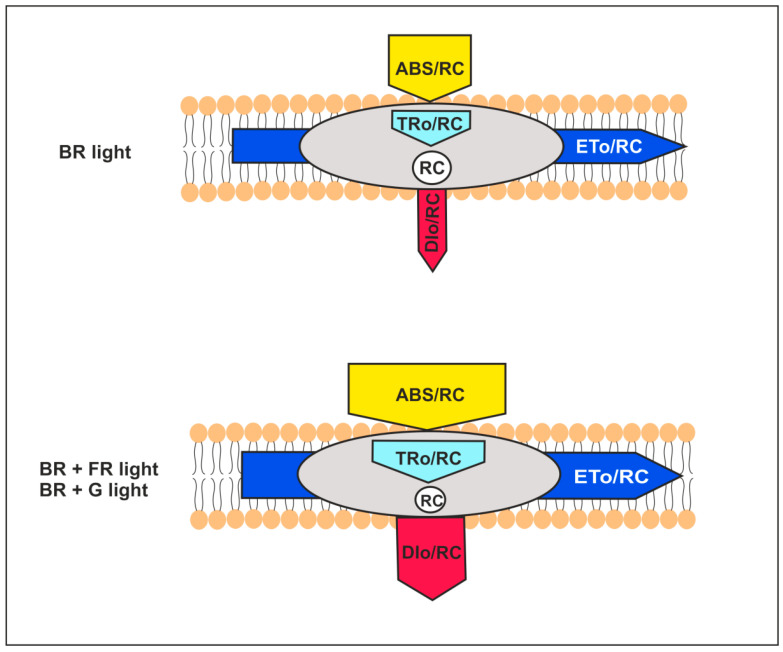
Pipeline membrane model for specific fluxes in PSII of *Gloeobacter violaceus* under different light conditions at the 30th day of the culture. The model demonstrates the specific energy fluxes per reaction center (RC): ABS/RC, TRo/RC, ETo/RC, and DIo/RC (for details see Table 3 and text). The value of each energy flux parameter is expressed by the appropriate adjustment of the corresponding arrow.

Among the various fluorescent data analyzed here, interesting changes were observed for parameters linked to energy fluxes in PSII. For day 30, we used the pipeline model to visualize the energy fluxes modified by the light environment (Figure 7). The width of the arrows reflects the values of selected energy flux parameters collected based on the data presented in Figure 6 and Supplementary Materials Table S1.

DIo/RC, a part of the flux of light excitation energy dissipated mainly as heat per RC and ABS/RC that is the measure of the average antenna size [46,51], increased on days 25 and 30 in all light conditions, in comparison to BR (control); an exception is the ABS/RC value on day 30 for BR + UVA (Figure 6 and Supplementary Materials Table S1). The model for day 30 indicates that *G. violaceus* exposed predominantly to BR + FR, and BR + G increased both the average antenna size and energy dissipation as indicated by elevated values of ABS/RC and DIo/RC, respectively. ABS/RC and DIo/RC were not changed in BR + UVA *G. violaceus* samples, while TRo/RC and ETo/RC values were increased for all light conditions on day 30 (Figure 6 and Supplementary Materials Table S1). Moreover, the increase in antenna size was related to the decrease in chl *a* content, while C-PC and C-PE in PBSs increased in BR + FR, BR + UVB, and BR + G samples. Most likely, the drop in the chl *a* content in RC induced changes related to the optimization of the *G. violaceus* light-harvesting system via an increase in antenna size (ABC/RC) based on the phycobiliproteins content in PBSs (Figure 2D–F, Figure 4C, Figure 6 and Figure 7).

#### 2.3.3. Principal Component Analysis (PCA)

**Figure 8:**
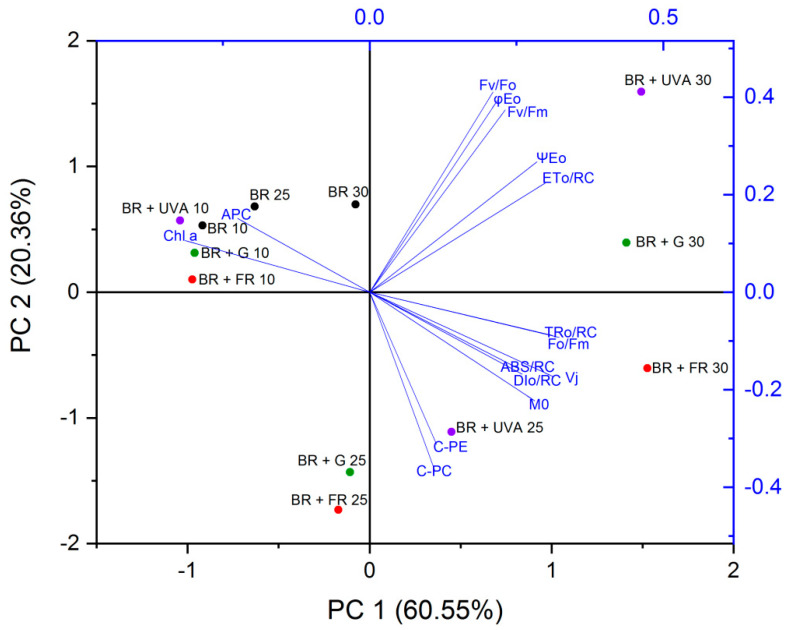
Principal Component Analysis (PCA) of the variability of the OJIP-test parameters and photosynthetic pigments in *Gloeobacter violaceus* from different days of culture (10th, 25th and 30th day) and light conditions: BR (control), BR + FR, BR + UVA, and BR + G. The abbreviations are described in Table 3 and Figure 9.

The results of the PCA analysis showed a positive correlation (r > 0.7) between chl *a* and APC, which were negatively correlated with the other tested phycobiliproteins (C-PC and C-PE), and photosynthetic parameters: TRo/RC, ABS/RC, DIo/RC, Vj, and M0 (Figure 8). Moreover, the highest values of chl *a* and APC were found in *G. violaceus* on the 10th day of culture, regardless of the spectral composition of light, and in the following phases of growth (day 25 and 30) in the cyanobacteria from BR light only (Figure 8). Additionally, a positive correlation between C-PC and C-PE (r > 0.96) was found. However, a low positive correlation (r > 0.4) between these two pigments (C-PC and C-PE) and some of the photosynthesis parameters, such as TRo/RC, ABS/RC, DIo/RC, Vj, and M0, was observed. The highest contents of C-PC and C-PE were found in the cultures grown under BR + UVA light. However, their levels decreased in the cultures from BR + FR and BR + G light on day 25 in comparison to the control (BR) (Figure 8). On the 30th day of culture, the highest values of TRo/RC, ABS/RC, DIo/RC, Vj, and M0 parameters were found in *G. violaceus* grown under BR + FR light compared to other cultures (Figure 6—30th day BR + FR). It was also observed that the photosynthetic parameters i.e.: Fv/Fo, Fv/Fm, ETo/RC, φEo, and ψEo, were negatively correlated with the other parameters and with photosynthetic pigments. However, Fv/Fo, Fv/Fm, ETo/RC, φEo, and ψEo showed a very strong positive correlation with each other (r > 0.9). It should be noted that their highest values were found in the cyanobacteria grown under BR + UVA and BR + G light on the 30th day of culture (Figure 8).

## 3. Discussion

The occurrence of cyanobacteria in various habitats indicates their high adaptability to changes in the environment. Light is an important factor influencing the proper growth of cyanobacteria. The light is absorbed by the photoreceptors, which combine light signals with oxygenic photosynthesis and other metabolic pathways [52,53,54]. In cyanobacteria, there are two specific aspects of the light-dependent processes. The first concerns the role of different wavelengths of light on the biosynthesis of PBS. The second feature refers to how cyanobacteria adjust light harvesting and the functioning of the photosynthetic apparatus to the light intensity and its wavelength [10,15,55,56]. 

### 3.1. The Spectral Composition of Light Affects the Growth of Gloeobacter violaceus

The colonization of limestone rock and alkaline substrates [11,57] and an unusual structure of the photosynthetic apparatus may suggest different preferences of *G. violaceus* for the spectral composition of light than other cyanobacteria living in typical aquatic environments. The first studies concerning *G. violaceus* and light conditions were carried out with the filters passing a specific light [31]. Currently, for research on the influence of monochromatic light on growth and development of plants, algae, and cyanobacteria, specially dedicated monochromatic lamps based on the LED technology are used [58]. Our research has shown that the spectral composition of light significantly affected the growth of *G. violaceus*, and its shape. In natural conditions, *G. violaceus* cells reach the size of 0.6–1.8 μm in diameter and 1.2–8.6 μm in length [5,31,59], and it is in the range reported in our studies (Table 1). According to Mareš et al. (2019), phylogenetically old cyanobacteria like *G. violaceus* usually have small dimensions, and the cells with a diameter ca. 2 μm represent a physiological limitation to packing entire thylakoid structures. Light-dependent changes in cyanobacterial shape have been reported previously [60]. For example, in the filamentous cyanobacterium, *Fremyella diplosiphon*, more spherical and shorter cells were observed in red light, whereas rectangular and longer cells were related to the growth in green light [61]. We can speculate that the more flattened and ellipsoidal *G. violaceus* cells in BR + G light are a form of adaptation to maximize the green light penetration through the cell and its harvesting by photosynthetic apparatus. 

### 3.2. Light Wavelengths Modify Pigments Content in Gloeobacter violaceus

PBSs, as a key light-harvesting structure, are not a part of the chl-binding proteins of PSII core but perform a crucial role in photosynthesis of cyanobacteria. It has been observed that the content of chls in algae and cyanobacteria decreases with the age of cells, as well as with the high light intensity [62]. When the light and thermal conditions are optimal for photosynthesis, the amount of organic compounds increases faster than the content of chl, and therefore the content of chl in the biomass decreases [63]. The previous studies showed that under red light (640 nm), PC and APC content was increased, while for the cells grown under green light (550 nm), the level of PC decreased but of PE increased [15]. It is believed that the light conditions affect the photoreceptors controlling the complementary chromatic adaptation (CCA) in cyanobacteria [10]. CCA photoreceptors absorb in the red and green range of photosynthetically active radiation (PAR) but demonstrate different responses to these light wavelengths. In red light, PC biosynthesis prevails, while in green light, PE production is dominant. Exposure of cells to natural sunlight, which is a mixture of different wavelengths of light (including green and red colors), causes the synthesis of phycobiliproteins with an intermediate content of PC and PE [10,31,64]. Here, we demonstrated different amounts of photosynthetic pigments depending on the spectral composition of light and the phase of *G. violaceus* growth (Figure 2). This indicates a light-dependent adaptation of *G. violaceus* photosynthesis. A clear role of the culture age in the level of phycobiliproteins (C-PC and C-PE) was also found. Their decreased content may suggest a slowdown of photosynthesis and cell growth as a result of cells competition for light [10]. Moreover, the pigment content may also vary depending on the phase of growth and the growth conditions as a result of reaching the limit of nutrients [10,65,66]. Another indicator of light-induced changes in the *G. violaceus* metabolism was the absorption spectrum of *G. violaceus* cultures (Figure 3). The obtained spectra confirmed the occurrence of main, previously characterized pigments and pigment-protein complexes: phycobiliproteins chl *a* and carotenoids [11,15,30,31,39,42]. 

### 3.3. The Activity of PSII in Gloeobacter violaceus Depends on the Spectral Region of Light

Previous studies have shown that the content of phycobiliproteins has also a significant impact on the fluorescence yield of PSII, the composition and organization of the antenna rods in cyanobacteria [2,3,67,68,69]. Measurements of chl *a* fluorescence are also used in cyanobacteria to monitor PSII efficiency in various stressful conditions [70,71,72,73,74,75,76]. To our knowledge, chlorophyll fluorescence transients are not sufficiently recognized in *G. violaceus*. A frequently used parameter to evaluate the functioning of the photosynthetic apparatus is Fv/Fm. In cyanobacteria, this parameter is much lower than in terrestrial plants or algae. This is due to the different structures of photosynthetic antennae (PBS) and the lack of chlorophyll *b* in most cyanobacteria. Bernát et al. (2012) showed that the Fv/Fm parameter ranged from 0.2 to 0.6 depending on the light intensity and temperature conditions in *G. violaceus*, and they are within the range of Fv/Fm described for *Synechococcus* sp. PCC 6301 and the values obtained in the present work (Figure 6) [27,68]. Moreover, the study of six cyanobacteria species showed that Fv/Fm (0.6–0.7) was higher in marine cyanobacteria with a low PBS to chl ratio in comparison to freshwater cyanobacteria (Fv/Fm 0.5–0.6), which had a higher PBS to chl ratio [77]. These varied results of the Fv/Fm parameter allow for observing how this parameter can differ depending on the strain and living conditions. Cyanobacteria can change the amount of energy transfer from PSII to PSI. This depends on the spectral composition of the light which affects the production of PBSs [78]. Blue light causes the extinction of energy in the PSI, while the G and FR lights generate energy without losses when moving from PBS to chl in RC. However, but the fastest transfer of energy was in the cells grown under yellow light [78]. In cyanobacteria, PETC depends on the spectral composition of light. For example, the PSII quenching analysis showed that energy captured by C-PE is transferred from the PBS to the PSI to keep the balanced electron transport when the *Calothrix* sp. PCC 7601 strain is grown under G light [8,79]. 

The general demonstration of the excitation energy fluxes per RC in PSII is shown in the pipeline model (Figure 7). In *Spirodela polyrhiza*, under Cr stress conditions, an increase in antenna size (ABS/RC) has been linked to a simultaneous decline in chl content and an increase in the energy flux used in PSII photochemistry (TRo/RC, ETo/RC) [80]. The increase in the average antenna size in *G. violaceus* is due to inactivation of some RCs linked to the depletion of chl *a* content in RCs, and that most likely is a result of the increasing size of PBSs, which is in line with increasing dissipation of total energy per RC (DIo/RC). However, unlike in *S. polyrhiza*, the specific energy fluxes per RC for trapping (TRo/RC) and electron transport (ETo/RC) in PSII of *G. violaceus* were increased. Additionally, it cannot be excluded that ETo/RC increased due to thermal activation of the dark reactions [46]. Moreover, in *S. polyrhiza*, the Cr-induced increase in ABS/RC has been related to the inhibition of growth [80]. In contrast to this, the enlarged antenna size of PSII in *G. violaceus* cultured in BR + FR and BR + G light did not change or even induce PSII efficiency (ψEo, φEo). In the same light conditions, the largest increase in biomass was observed (Figure 1). It cannot be excluded that the increased electron transport (ETo/RC, ψEo, φEo) also affects PSI activity and the generation of metabolic energy via cyclic electron transport, especially under BR + FR light conditions. The obtained data indicate that the spectral properties of light strongly affect PETC in PSII of *G. violaceus*, which is clearly different than in plants. 

However, an additional source of energy needed for growth might also be derived from other light-dependent metabolic energy generating systems. The largest biomass growth was found for cyanobacteria grown under BR + FR and BR + G light. It suggests that *G. violaceus* photosystems absorb in the red and green PAR regions, and the production of ATP, driven by proton (H+) gradients, is also established by the cell membrane-located GR [42,81]. Several studies have documented that the presence of rhodopsin in some microorganisms supports their growth and/or survival under stress conditions, as well as participates in various physiological processes [82,83,84,85,86,87,88,89]. Thus, rhodopsin can compensate for the poor production of energy from PETC by creating an additional proton gradient in the periplasm. According to this model, *G. violaceus* transforms light energy into a proton driving force using phycobilin and GR [42]. Vogt et al. (2013) described that the function of rhodopsin can maintain a temporary membrane tension when photosynthesis is not based on chl [90]. It allows for assuming that the GR-based proton pump was activated in *G. violaceus* grown under BR + FR and BR + G light. 

## 4. Materials and Methods

### 4.1. Cyanobacteria

The strain *Gloeobacter violaceus* (CCALA 979) was purchased from the Culture Collection of Autotrophic Organisms (CCALA) of Institute of Botany from University in Prague (Czech Republic). This strain is an ecotype of the reference strain *G. violaceus* PCC 7421 [33].

### 4.2. Growth Conditions

*G. violaceus* was cultivated on the BG11 medium [91] in 500 mL Duran bottles (with 400 mL working volume). The bottles and medium were sterilized by autoclaving (121 °C, 21 min). After autoclaving, the medium was adjusted to pH = 7.5. The bottles were bubbled with air using a membrane aquarium pump (SERA Air 550 R Plus, Heinsberg, Germany). For the culture, 400 mL of medium was used, to which 16 mL of the cyanobacterial suspension was added. The cultures were grown for 30 days at 25 ± 1 °C at a photoperiod of 16/8 h (light/dark). 

### 4.3. Light Sources

**Figure 9:**
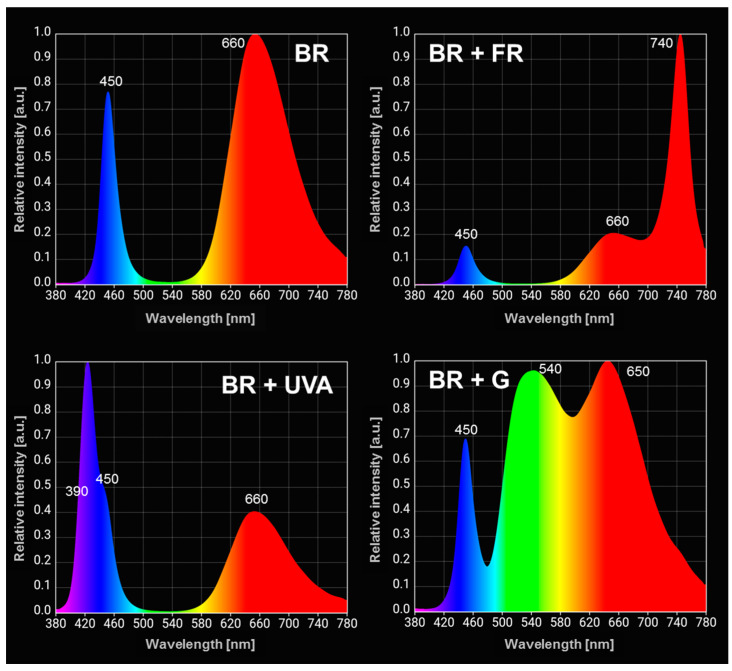
The spectral composition of light-emitting diodes (LEDs) matrix used during the culture of *Gloeobacter violaceus*. BR—blue and red light (control); BR + FR—blue and red light enriched with the far-red light; BR + UVA—blue and red light with the addition of UV-A light; BR + G—blue and red light with the addition of the green light. The maxima for the wavelengths are shown.

Light sources based on light-emitting diodes (LEDs) with different spectral light compositions were used. An LED lamp with a maximum wavelength in the blue (440 nm) and red (660 nm) region of the spectrum was applied as a control light (BR). The BR lamp was the basis for the construction of lamps with the addition of a different wavelength of light. The next lamps consisted of the BR lamp with the addition of a wavelength in another region of the spectrum, i.e., the BR + far-red (FR) lamps with an additional wavelength in the FR spectrum region (740 nm), BR + ultraviolet A (UVA) with the UVA spectrum region (390 nm), and BR + green (G) with the green region (550 nm) of visible light. The spectral composition of individual LED lamps is shown in Figure 9. The light intensity was adjusted to the level of 70 µmol photons m^−2^ s^−1^, regardless of the spectral composition of the LED lamps. 

The light intensity was measured in three various places at the surface of culture bottles using the Asensetek Lighting Passport Spectrometer (New Taipei City, Taiwan).

### 4.4. Determination of Biomass Growth

The growth rate of *G. violaceus* biomass was determined based on the optical density (OD) of the culture. OD was measured as the absorbance of the cell suspension at 730 nm on a microplate reader (Synergy 2, BioTek, Winooski, VT, USA). Medium BG11 was used as a blank.

### 4.5. Size of Gloeobacter violaceus Cells

The size of the cells was measured using a scanning electron microscope (SEM), which overview is presented in Figure S2. Minor and major cell axes were measured to visualize the shape of the cells. The minor cell axis is the perpendicular length between the cell cavity and its end (a), while the major cell axis is the length along the cell (b) (Table 1). The ratio of axis a to axis b indicates whether the cell has the shape close to a sphere (ratio ~ 0.8–1.0) or an ellipse (ratio < 0.7). The cell shape model was performed by CorelDraw 2020 program.

### 4.6. Content of Photosynthetic Pigments in the Gloeobacter violaceus Culture

#### 4.6.1. Chlorophyll and Carotenoids

The content of chlorophyll a (chl *a*) is only presented due to the absence of chlorophyll *b* (chl *b*) for most of the cyanobacteria [92]. According to Herrmann and Gehringer (2019) [38] *G. violaceus* possess only chl *a*. Determination of chl *a* and carotenoid (car) contents in the *G. violaceus* biomass was performed according to the method of Leu and Hsu (2005) [93] by using the equations proposed by Liechtenthaler (1987) [94].

chl *a* =16.72 × A665 − 9.16 × A652 (µL·mL^−1^) (1)

car = (1000 × A470 − 1.63 × chl *a*)/221 (µL·mL^−1^) (2)

A total of 2 mL of pure methanol was added to the centrifuged pellet of the sample. The samples were homogenized for 5 min in an ultrasonic bath, incubated at 60 °C for 40 min, and then transferred to 0 °C for 15 min. Then, the contents of chl *a* and car were determined spectrophotometrically based on absorption values at 470 (A470), 665 (A665) and 652 (A652) nm. The content of the pigments was normalized into OD730 nm of the culture on a given day.

#### 4.6.2. Phycobiliproteins

To extract phycobiliproteins from *G. violaceus*, the biomass was lyophilized for 48 h and then the cells were mechanically disrupted in the tissulizer (Retsch, MM400). A total of 2 mL of sodium phosphate buffer (0.1 mM, pH 7) [95] was added to the biomass and homogenized for 5 min in an ultrasonic bath. After this, the samples were centrifuged for 15 min at 4 °C at 4300× *g* (Hettich). The content of phycobiliproteins: phycocyanin (C-PC), allophycocyanin (APC), and phycoerythrin (C-PE), in the supernatant was determined by the absorbance at wavelengths: 620 (A620), 652 (A652), and 562 (A562) nm [96]. The content of phycobiliproteins was normalized into the OD730 nm of the culture.

Phycobiliproteins concentration was calculated according to the equations by Bennett and Bogorad (1973) [96]:

C-PC = (A620 − 0.474 × A652)/5.34 [mg mL^−1^] (3)

APC = (A652 − 0.208 × A620)/5.09 [mg mL^−1^] (4)

C-PE = (A562 − (2.41 ∗ C − PC) − (0.849 × APC))/9.62 [mg mL^−1^] (5)

### 4.7. Absorption Spectroscopy

The absorption spectra of the *G. violaceus* suspension and extracted photosynthetic pigments were determined with a JASCO V-630 spectrophotometer UV-VIS with Spectra Manager™ CFR software. The cells were collected directly from the culture, while the photosynthetic pigments (phycobiliproteins, chlorophyll, and carotenoids) were extracted as described above. The measurement was performed in a quartz cuvette in the range of 375 to 800 nm with a scanning speed of 400 nm/min. Spectra deconvolution was performed with the normalization to the largest peak (628 nm) and then the Lorenz components were fitted. For the spectra analyses, OriginLab 2020 software was used. 

### 4.8. Photosystem II Activity

PSII photochemical efficiency of *G. violaceus* was determined by the fast fluorescence method. The chl *a* fluorescence parameters were measured using a Handy Plant Efficiency Analyzer apparatus (Handy PEA, Hansatech Instruments, King’s Lynn, UK) with an adapter used for liquid samples at room temperature. The sample was illuminated with a red light saturating flash of 3500 μmol photons m^−2^ s^−1^ with a 637 nm peak wavelength. A total of 2 mL of cell suspension—collected directly from the cultures—was added to the measuring cuvette. The measurements were performed after 6 min adaptation of the sample to the darkness. Adaptation time was optimized experimentally before the start of the experiments. Kinetic fluorescence parameters were recorded within the range of 10 µs to 1 s. The analyses of the relationship between the structure and function of PSII and the assessment of the vitality of *G. violaceus* were based on the OJIP test [7,8,62,76,97,98]. The list of analyzed fluorescence parameters is shown in Table 3 [46,99,100,101]. The fluorescence parameters and OJIP curves were normalized to the content of chl *a*.

#### Principal Component Analysis

To show the more complex correlation between chl *a* fluorescence parameters and photosynthetic pigments in the cultures of *G. violaceus* grown under different spectral compositions of light, a principal component analysis (PCA) was performed. OriginLab software was used to draw the charts.

### 4.9. Statistical Analysis

The results presented in the study represent the means of four independent biological repetitions. The comparison of the significance of differences in mean values between objects was performed using the Tukey’s HSD test procedure at the level of significance: *p* ≤ 0.0001 (***), *p* ≤ 0.001 (**), and *p* ≤ 0.01. The statistical analysis was performed using GraphPad v. 7.01 and OriginLab 2020 programs.

## 5. Conclusions

The study emphasized a significant effect of FR and G light on the growth of the *G. violaceus* cultures. Based on the quantitative analyses of phycobiliproteins content and PETC carried out in this study, it can be assumed that both the spectral composition of light and age of the cyanobacterial culture affect the level of C-PC and C-PE in the PBS rode. The contents of C-PC, C-PE, and APC are related to the light wavelength absorbed by phycobiliproteins. Moreover, the modification of the shape of *G. violaceus* cells under G light suggests a morphological adaptation of this cyanobacteria species to the changing light environment. Most likely, BR + FR and BR + G light also influence the PBS-based antenna, PSII efficiency, and GR-related generation of metabolic energy. For further studies, deeper structural analyses of PBS isolated from *G. violaceus* grown under different spectral compositions of light are needed.

## Figures and Tables

**Table 1:** *Gloeobacter violaceus* cell sizes and shapes, which were obtained on the 30th day of the culture growth under different light conditions. The data represent means ± SD (*n* = 10). The star indicates statistical significance from BR light (control) at the level *p* < 0.05 (*).

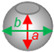	The Spectral Composition of Light			
	BR (Control)	BR + FR	BR + UVA	BR + G
**Minor axis (a) (µm)**	0.9 ± 0.1	0.8 ± 0.1	1.0 ± 0.1	0.9 ± 0.2
**Major axis (b) (µm)**	1.3 ± 0.2	1.0 ± 0.1	1.3 ± 0.2	1.5 ± 0.3
**Ratio a:b**	0.7 ± 0.1	0.8 ± 0.1	0.8 ± 0.1	0.6 ± 0.1 *
**The model of the cell shape**	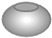	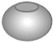	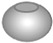	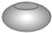

**Table 2:** Identification of photosynthetic pigments based on the area of each peak of the absorption spectrum after deconvolution of the *Gloeobacter violaceus* culture spectrum at day 30 under different light spectral composition. Identification of peaks for each of the pigments was based on the position of the peaks found in the literature [15,30,31,41,42].

Component (Photosynthetic Pigments)	Peak Maximum Wavelength (nm)	BR	BR + FR	BR + UVA	BR + G
		Peak Area (a.u.)			
**chl *a***	440	7	8	6	13
**car**	475	-	-	-	8
**R-PE, car, PUB**	500	6	10	8	13
**C-PE, GR, B-PE**	548	11	8	9	8
**C-PE, R-PE**	565	22	15	19	10
**C-PC**	628	77	73	78	58
**chl *a*, APC**	680	7	10	10	22

**Table 3:** The description of chl *a* fluorescence parameters used in these studies [97,101].

Energy Flow Rates	
**Fo/Fm**	quantum yield baseline, it is used as an indicator of the physiological state (state change index);
**Fv/Fm**	a value that is related to the maximum quantum yield of PSII;
**Fv/Fo**	a value that is proportional to the activity of the water-splitting complex on the donor side of the PSII;
**Vj**	relative variable fluorescence at phase J of the fluorescence induction curve;
**M0**	the approximated initial slope of the fluorescent transient, which is related to the rate of closure of reaction centers;
**Specific Energy Fluxes**	
**ABS/RC**	the antenna size of active PSII per reaction center (RC) and light absorption energy flux (for PSII antenna chlorophylls);
**DIo/RC**	total energy dissipated per reaction center (RC) as heat, fluorescence, and energy transfer to PSI;
**TRo/RC**	trapping flux leading to Q_A_ reduction per reaction center (RC);
**ETo/RC**	the electron flux transferred per active reaction center (RC);
**Quantum Yields**	
**ψEo (Ψo)**	probability (at time 0) that a trapped exciton moves an electron into the electron transport chain beyond Q_A_^−^;
**φEo**	the quantum efficiency of electron transfer from Q_A_^−^ to plastoquinone.

## Data Availability

Data sharing not applicable.

## Supplementary Materials

The following are available online at https://www.mdpi.com/article/10.3390/ijms22084021/s1, Figure S1: The 30-day growth of the cultures in Erlenmeyer flasks under different light conditions, Figure S2: SEM overview of *Gloeobacter violaceus* culture grown under BR light (A), BR + FR light (B), BR + UVA light (C), and BR + G light (D), Table S1: Chlorophyll *a* fluorescence parameters of *Gloeobacter violaceus* from different spectral compositions of light and days of culture.

## Abbreviations

APC	allophycocyanin
B-PE	B-phycoerythrin
C-PC	phycocyanin
C-PE	phycoerythrin
car	carotenoids
chl	chlorophyll
chl *a*	chlorophyll *a*
chl *b*	chlorophyll *b*
GAF	cGMP-specific phosphodiesterases/cyanobacterial adenylate cyclases/FhlA domain
GR	gloeorhodopsin
PAS	Per/ARNT/Sim domain
PHY	phytochrome-specific domain
PUB	phycourobilin
RC	reaction center
R-PE	R-phycoerythrin
