# Supplementary material for: The Role of Selected Wavelengths of Light in the Activity of Photosystem II in Gloeobacter violaceus
Source: Int J Mol Sci. 2021 Apr 13;22(8):4021. doi: 10.3390/ijms22084021 (PMC8069770; doi:10.3390/ijms22084021)
Supplement: Supplementary file 1 [file ijms-22-04021-s001.pdf]

Supplementary Materials

The role of selected wavelengths of light in the activity of photosystem II in *Gloeobacter violaceus*

Monika Kula-Maximenko[1], Kamil Jan Zieliński [1], Ireneusz Ślesak [1*]

[1]*The Franciszek Górski* Institute of Plant Physiology, Polish Academy of Sciences, Niezapominajek 21, 30-239 Kraków, Poland

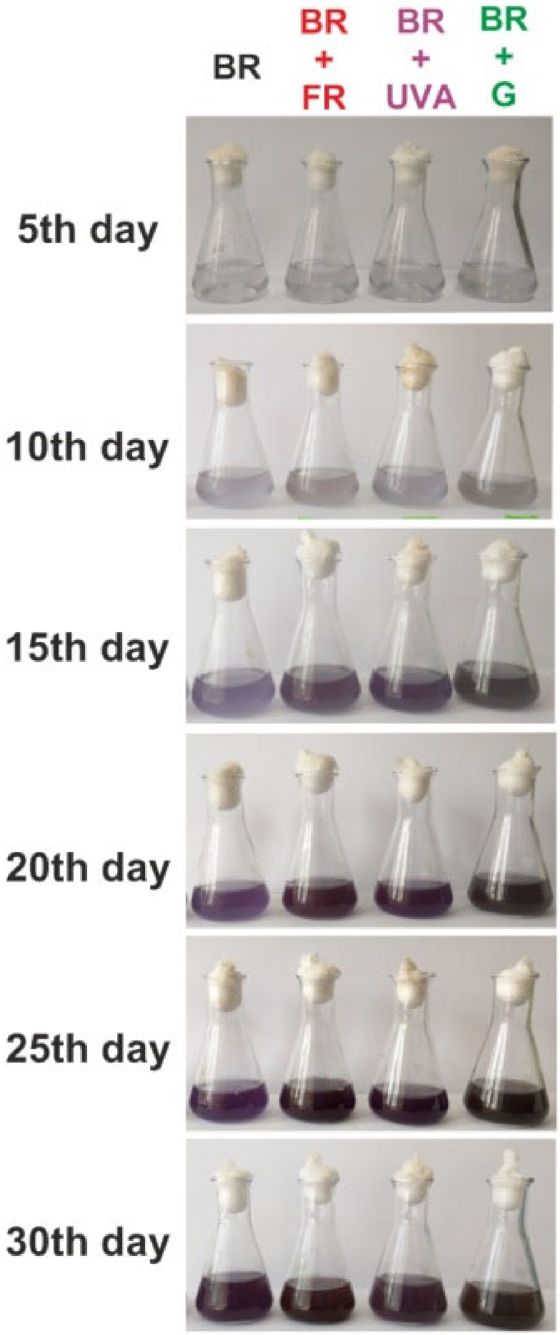

Figure S1. The 30-day growth of the *Gloeobacter violaceus* cultures in Erlenmeyer flasks under different light conditions.

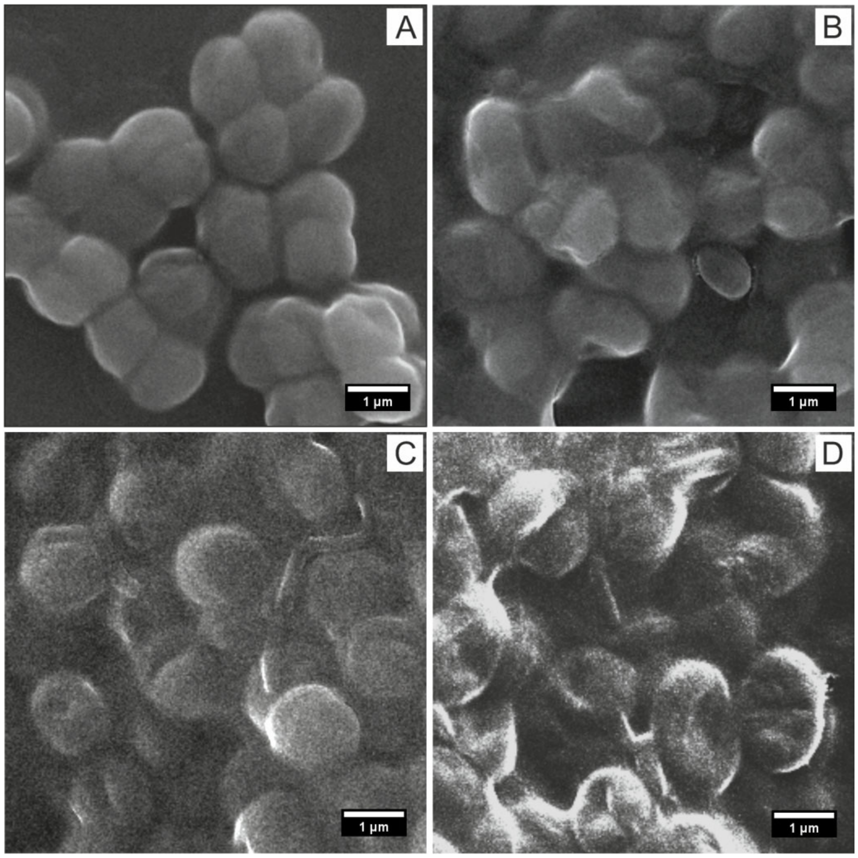

Figure S2. SEM overview of *Gloeobacter violaceus* culture grown under BR light (A), BR + FR light (B), BR + UVA light (C), and BR + G light (D).

Table S1. Chlorophyll *a* fluorescence parameters of *Gloeobacter violaceus* from different spectral compositions of light and days of culture. The data presented are the average values from 8 independent repetitions for each object. The values marked with different letters are significantly different according to light conditions using a one-way ANOVA and Tukey's HSD test procedure at the level of significance $p \leq 0.05$.

Parameter	Spectral composition of light											
	BR	BR +FR	BR + UVA	BR + G	BR	BR + FR	BR + UVA	BR + G	BR	BR + FR	BR + UVA	BR + G
	10th days of culture				25th days of culture				30th days of culture			
**Fo/Fm**	0.605^{b}	0.598^{b}	0.566^{c}	0.680^{a}	0.772^{d}	1.140^{c}	1.239^{b}	1.329^{a}	0.973^{c}	2.032^{a}	1.716^{b}	1.810^{b}
**Fv/Fm**	0.171^{a}	0.126^{c}	0.158^{b}	0.137^{c}	0.173^{b}	0.072^{c}	0.208^{a}	0.046^{c}	0.217^{a}	0.025^{b}	0.380^{a}	0.260^{a}
**Fv/Fo**	0.219^{a}	0.153^{b}	0.203^{a}	0.165^{b}	0.212^{a}	0.076^{b}	0.244^{a}	0.048^{b}	0.266^{a}	0.026^{b}	0.487^{a}	0.328^{a}
**Vj**	0.558^{ab}	0.534^{b}	0.506^{c}	0.596^{a}	0.623^{b}	1.060^{a}	1.072^{a}	1.128^{a}	0.783^{b}	1.587^{a}	1.282^{a}	1.349^{a}
**ABS/RC**	8.15^{c}	9.36^{b}	7.53^{c}	10.84^{a}	11.25^{d}	42.79^{b}	21.65^{c}	60.79^{a}	13.58^{b}	47.60^{a}	26.53^{b}	75.22^{a}
**DIo/RC**	6.36^{c}	7.73^{b}	5.90^{c}	9.03^{a}	9.19^{c}	40.26^{b}	18.57^{c}	57.61^{a}	11.11^{b}	47.36^{a}	22.46^{b}	71.67^{a}
**TRo/RC**	1.79^{a}	1.63^{b}	1.63^{b}	1.81^{a}	2.06^{c}	2.53^{b}	3.08^{a}	3.18^{a}	2.47^{c}	5.24^{a}	4.06^{b}	3.54^{b}
**ETo/RC**	0.501^{a}	0.430^{a}	0.493^{a}	0.490^{a}	0.702^{a}	0.316^{b}	0.798^{a}	0.693^{a}	0.847^{b}	1.301^{a}	1.522^{a}	1.175^{a}
**M0**	1.29^{ab}	1.20^{b}	1.14^{bc}	1.32^{a}	1.36^{c}	2.21^{b}	2.28^{ab}	2.60^{a}	1.62^{b}	3.94^{a}	2.54^{b}	2.37^{b}
**ψEo**	0.217^{a}	0.191^{a}	0.219^{a}	0.221^{a}	0.32^{b}	0.152^{c}	0.406^{a}	0.292^{b}	0.407^{b}	0.470^{b}	0.814^{a}	0.721^{a}
**φEo**	0.048^{a}	0.033^{b}	0.048^{a}	0.037^{b}	0.059^{a}	0.009^{b}	0.055^{a}	0.006^{b}	0.075^{b}	0.052^{b}	0.175^{a}	0.144^{a}
